# Promoting the Physical Activity of Older Adults in Institutional Long‐Term Care: A Mixed‐Method Case Study

**DOI:** 10.1111/opn.70053

**Published:** 2025-11-17

**Authors:** Noora Narsakka, Riitta Suhonen, Johanna Finskas, Minna Stolt

**Affiliations:** ^1^ Department of Nursing Science University of Turku Turku Finland; ^2^ Turku University Hospital and City of Turku Welfare Division Turku Finland; ^3^ Wellbeing Services County of Satakunta Pori Finland

**Keywords:** environment, health promotion, long‐term care, nursing care, nursing homes, older adults, physical activity

## Abstract

**Introduction:**

Older adults living in institutional long‐term care benefit from engaging in physical activity adapted to their functioning. Despite evidence of solutions to promote physical activity, recurrent evidence shows that older adults spend their time sedentary. More in‐depth knowledge is needed about the current state of promoting the physical activity of older adults in institutional long‐term care for improved practice in the future. We aimed to increase the understanding of older adults' physical activity promotion in institutional long‐term care by investigating how, how much and by whom older adults' physical activity is promoted.

**Methods:**

This is a concurrent mixed‐method case study using data from a larger research project performed in an institutional, full‐time, long‐term care unit in Finland. Thirteen older adults and 12 staff members participated. Data were collected through focus groups, interviews, patient record transcripts and actigraphy between May and October 2023. A mixed‐method analysis was conducted using the framework ‘Following a thread’. Separate analyses of datasets were conducted, including analyses of qualitative and quantitative data using reflexive thematic analysis and descriptive statistics. Analytical questions were identified and further explored using all datasets to synthesise findings.

**Results:**

Four themes were developed: (1) lack of physical activity, (2) plans for physical activity promotion, (3) nurses' role in activity promotion and (4) accessibility and freedom of movement.

**Conclusion:**

Current activity promotion is not sufficient for older adults to achieve the benefits of physical activity for their health and functioning. Improvements are needed in delivering sufficient physical activities. Nurses' role in activity promotion should be developed to include care‐integrated activities, spontaneous and organised activities and instrumental activities of daily living for older adults. Interprofessional work to promote activity could be used more. Stimulating elements in the physical environment and increasing freedom of movement could produce improvements in physical activity. Improvements in activity promotion can potentially be achieved with simple strategies and low additional costs.

**Summary**

- What does this research add to existing knowledge in gerontology?
  ○ Generates evidence on physical activity and its promotion involving older adults in institutional long‐term care with advanced functional decline, who are often excluded from studies.
  ○ Produces in‐depth knowledge of the physical activity promotion of older adults in institutional long‐term care, highlighting contradictions between planned physical activity promotion and its enactment.
- What are the implications of this new knowledge for nursing care for and with older adults?
  ○ Physical activity promotion requires more attention as a part of fundamental nursing practice, with various benefits for care‐dependent older adults living in institutional long‐term care.
  ○ Nurses' role in physical activity promotion should be developed to incorporate it as part of everyday practice.
  ○ Various simple, low‐cost strategies could potentially be used to improve physical activity promotion involving different professionals and family members.
- How could the findings be used to influence practice, education, research, and policy?
  ○ To improve the physical activity promotion of older adults, multicomponent physical activity interventions that can be modified to heterogeneous populations and contexts should be developed and tested involving stakeholders.
  ○ Nursing professionals would benefit from education about the benefits of physical activity for older adults and opportunities to develop their own practices to improve the physical activity promotion of older adults.
  ○ To achieve a sufficient level of promotion of older adults' activity in institutional long‐term care, organisations could engage in systematic evaluations of their policies, practices and the care environment.

## 1 Introduction

Physical activity promotion is vital to counteracting the current trend of sedentary lifestyles of older adults in institutional long‐term care. Currently, the proportion of the ageing population is increasing worldwide (Organisation for Economic Co‐operation and Development [OECD] 2021). At the same time, those living in institutional long‐term care settings are mostly aged 75 and above. Increasingly, they experience complex multimorbidity and disability (Barker et al. 2020), needing personal assistance to meet their basic needs, conduct activities of daily living and be mobile (Palese et al. 2016). Of them, more than 70% experience dementia (Edgren et al. 2024; Lane et al. 2017). Dementia leads to impairments in cognitive and physical functioning; changes in mood, motivation and behaviour; and restrictions in the capability to conduct activities of daily living and to live alone safely (World Health Organization and Alzheimer's Disease International 2012). Despite multimorbidity and disability, older adults benefit from engaging in physical activity adapted to their functioning. Being physically active means engaging in movement that is produced by skeletal muscles and results in energy expenditure (Caspersen et al. 1985). It is vital to prevent various somatic health conditions and depression, falls, further decline of functioning and progress of dementia (Cunningham et al. 2020). Furthermore, being physically active maintains independence in activities of daily living (Cunningham et al. 2020) and prevents further increase in care dependency (den Ouden et al. 2015).

Recommendations have been given for the levels of physical activity to achieve benefits for health and functioning for older adults living in the institutional setting. According to them, older adults should engage in moderate‐intensity activity twice a week for 35–45 min/session, conducting endurance, muscle strength, balance and coordination exercises, as well as engage in few‐minute bouts of physical activity 3–4 times a day (de Souto Barreto et al. 2016; Peyrusqué et al. 2023). According to evidence, these recommendations are not met in institutional long‐term care; most conducted activity is light‐intensity activity (Mc Ardle et al. 2021) and consists mostly of activities of daily living and mobility within the facilities (den Ouden et al. 2015; Hahn et al. 2023). Also, increasing light‐intensity activity may be beneficial. It can improve physical functioning (Baldelli et al. 2021) and activities of daily living (McArthur et al. 2024). However, older adults in institutional long‐term care spend most of their time sedentary—85% and 92% of the day (den Ouden et al. 2015; Hahn et al. 2023; Liu et al. 2020; Parry et al. 2019; Shi et al. 2024).

Individual‐level factors of older adults are associated with physical activity, including functioning (Jepson et al. 2023; Shi et al. 2024), motivation, attitudes (Maurer et al. 2019), self‐efficacy for and perceived benefits of physical activity (Shi et al. 2024) and fear of falling (Benjamin et al. 2014). However, considering the advanced functional decline of the population, factors beyond the individual are emphasised when conducting physical activity. These include features of the environment—such as accessibility and stimulating features—and a suitable social environment and cultural context (Bowes et al. 2022; Narsakka et al. 2022; Wylie et al. 2023). Promotion of physical activity by professionals during care activities is vital for this population. Professionals should, for example, support older adults' independence in activities of daily living and deliver exercise and walking activities (Hirt et al. 2024).

As presented above, there is evidence about factors associated with older adults' physical activity and strategies that can be used in activity promotion. Still, there seems to be a research–practice gap in implementing this evidence. Notably, many studies testing interventions (McArthur et al. 2017; Wylie et al. 2023) or reporting older adults' activity levels (Liu et al. 2020; Parry et al. 2019; Shi et al. 2024) have excluded persons with advanced cognitive or physical impairments. This hampers the use of the evidence or understanding the current state of physical activity and its promotion in these settings. Therefore, more in‐depth knowledge is needed about the current state of promoting the physical activity of older adults in institutional long‐term care for improved practice in the future.

To increase understanding of older adults' physical activity promotion in institutional long‐term care, we conducted a mixed‐method case study in one institutional long‐term care unit, investigating the research question:

How, how much and by whom is older adults' physical activity promoted in institutional long‐term care?

## 2 Materials and Methods

### 2.1 Design and Theoretical Underpinnings

The study was conducted as a concurrent mixed‐method case study (Creswell and Plano Clark 2018). We used data from a larger action research study aimed at increasing older adults' physical activity through changes in care practices and environment. In the present study, we used data collected before the implementation of changes; therefore, presenting the baseline of activity promotion in the unit. The integration of qualitative and quantitative findings allowed us to generate deeper insights and advance the current state of knowledge beyond what could be achieved through standalone methods. Our theoretical worldview was pragmatism, which identified a mixed‐method approach as a tool for integrating perspectives and approaches to best address our research question (Johnson et al. 2007). Furthermore, the theoretical basis for our work was Lawton's (1989) environmental press theory, which states that the quality of the environment becomes increasingly important for older adults with decreasing functioning. The quality of the environment we identified consists of the physical (concrete, material), social (persons and their relations) and symbolic (norms, values, culture) dimensions of the environment (Kim 2010).

### 2.2 Study Setting, Recruitment and Participants

We conducted the study in a full‐time institutional long‐term care unit in Finland, where it is provided on the basis of medical or safety‐related care needs that cannot be met in other settings. In Finland, approximately 4% of individuals aged 65 and over and 8% of individuals aged 75 and over reside in institutional long‐term care (Terveyden ja hyvinvoinnin laitos (THL) [National Institute for Health and Welfare] 2024). In 2022, their average age was 85 years. Their need for care to survive in activities of daily living was high. Ninety percent of residents experienced cognitive decline, with 70% having a formal diagnosis of dementia (Edgren et al. 2024).

Approximately 45% of long‐term care is provided by the public sector in Finland (Mielikäinen and Kuronen 2022). Our recruitment was purposive, approaching both public and private sector units and finally recruiting a public sector unit. The participating unit was located in a larger facility with several smaller closed units and shared facilities for all units, such as a library, a restaurant, a gym and a banquet hall. Residents could not exit independently from their living unit and could use the facilities outside of their living unit with staff or family members.

In institutional long‐term care units in Finland, 80% of staff members are licensed practical nurses with a vocational education of 180 European Credit Transfer and Accumulation System (ECTS) credits and 10% are registered nurses with a bachelor's degree of 210 ECTS credits. Rehabilitation professionals account for approximately 2% of staff, having a 210 ECTS bachelor's education (Kehusmaa and Alastalo 2021). In the participating unit, care was provided by 12 nursing staff members working in the unit daily, including practical and registered nurses and nursing aides. The proportion of rehabilitation professionals working in the unit was 8%; one physiotherapist worked in the unit 5 days a week, and one occupational therapist worked 1 day a week. In addition, social workers and cultural instructors (specialising in organising, guiding and developing cultural activities) at the facilities had time allocated to older adults approximately 3 days a week.

Everyone living or working in the unit and capable of communicating in the Finnish language was invited to participate. Altogether, 25 persons participated in the present study: 13 older adults living in the unit and 12 staff members working in the unit. Older adults' mean age was 84 years (range 65–102), and 62% of them were women. Of the 13 older adults, 8 (62%) walked independently with or without an aid, 2 (15%) ambulated independently with a wheelchair, 2 (15%) ambulated actively with an aid supported by a staff member and 1 (8%) ambulated passively with an aid supported by a staff member. Staff members were practical nurses (*n* = 6), registered nurses (*n* = 2), physiotherapists (*n* = 1), occupational therapists (*n* = 1) and head nurses (*n* = 2). They were all women, and their mean age was 40 years (range: 21–53).

### 2.3 Data Collection

We used focus groups, interviews, patient record transcripts and actigraphy to collect data (Table 1). NN collected qualitative data through focus groups and interviews with professionals working in the unit. For this purpose, we used a thematic interview guide that included questions on (1) how the actions of the different professional groups, family members and volunteers were related to the physical activity of older adults, (2) older adults' activities, content and amount and (3) how environmental factors were related to older adults' activity. We developed the interview guide with the help of the co‐researchers of the action research project based on earlier research evidence. The data were collected in Finnish. The focus group and the interviews were recorded and lasted 1.5–2.5 h. JF transcribed the data verbatim.

**TABLE 1:** Data collection in the study.

Objective of data use	Data collection method	Participants
Describing participants' perspectives on older adults' physical activity and physical activity promotion	Focus group	Nurse (*n* = 8)
	Individual interviews	Physiotherapist (*n* = 1) Occupational therapist (*n* = 1) Head nurse (*n* = 2)
Summarising older adults' physical activities and delivery of physical activities	Patient record transcripts related to physical activity of older adults	Older adult (*n* = 13)
Measuring level and intensity of older adults' physical activity	Actigraphy (Fibion SENS Motion)	Older adult (*n* = 13)

Quantitative data collection included physical activity measurements. Physical activity was measured using the Fibion SENS Motion (Fibion Inc. 2020) activity tracking system. Each participant wore a small waterproof activity tracker on their thigh to measure activity on seven consecutive days 24 h a day. These data were analysed in 5‐s intervals, with each segment classified into a specific activity category. The categories were light intensity activity, including standing (being upright, only minor movements), sporadic walking (light intensity walking intervals below 3–9 s or cycling intervals below 1 min) and continuous walking (light intensity walking for at least 5–10 s continuously), and moderate and high intensity activity, including fast walking (moderate intensity walking for at least 3–9 s continuously), cycling (cycling leg movement for at least 1–2 min) and vigorous activity (high‐intensity walking activity/running for at least 3–9 s continuously). In addition, we retrieved patient record information for older adults' physical activity from the patient records for the same 7‐day period. The data were collected between May and October 2023. Patient records were retrieved in March 2024.

### 2.4 Data Analysis

We used Moran‐Ellis et al.'s (2006) framework, called following a thread, which allows the analytical integration of data obtained with mixed methods. Using the framework, first separate analyses of datasets were conducted identifying analytical questions for further exploration. These questions (*threads*) were then iteratively investigated (*followed*) using all datasets to synthesise findings. Table 2 shows an example of the analysis process.

**TABLE 2:** Example of the analysis process ‘following a thread’.

Analysis phase	Purpose of phase	Example of analysis
First phase	Conducting separate analyses for each dataset to identify analytical questions for further exploration	Theme developed using thematic analysis for interview and focus group data: *Nurses' work is the most important (to promote older adults' physical activity)*. Based on the developed theme, a new question was chosen for further exploration: How much and how do nurses promote older adults' physical activity in light of the results of all datasets?
Second phase	Iteratively investigating identified analytical questions by using all datasets (following a thread), and creating a data repertoire	Patient record transcript results were used to investigate how much physical activities nurses organised and how much physical activity these activities resulted in. These results were compared to the actigraphy‐measured activity levels of older adults. All data relating to the question under investigation were collected together using a word sheet. Interpretations were written down. Using the results of all datasets, nurses' activity promotion consisted mostly of supporting independence in activities of daily living.
Third phase	Synthesising findings of threads to answer the original research question	New themes were developed to answer the research question. For the nurses' role in activity support, a new theme was developed based on the iterative mixed‐method analysis process: *Nurses' role in activity promotion*.

NN conducted the analysis. In the first phase, we analysed each dataset separately using appropriate methods and formulated analytical questions. This included three steps (A–C). (A) For interview and focus group data, we used reflexive thematic analysis (Braun and Clarke 2022). We read and coded inductively the data in NVivo 1.7.1 using semantic and latent approaches. Then, we exported the codes to Word and formulated themes looking for similarities and differences and an overall story within the data to answer the research question. We took notes to facilitate the process. We formulated four themes in the analysis and wrote initial descriptions of them. (B) We conducted analyses of the patient record transcript and actigraphy data. Using an Excel spreadsheet, the patient record transcripts were categorised according to the activities delivered and the persons delivering the activities and quantified. Quantitative data were analysed using descriptive statistics, calculating frequencies, means, medians and proportions. The normality of the data distribution was evaluated by examining histograms. Data analyses were conducted using SPSS version 29.0.0.0. (C) Using the separate results of all analyses we identified analytical questions for further exploration because of their relation to the original research question or because of their resonance in several datasets. We formulated five analytical questions to investigate.

In the second phase, we addressed the identified analytical questions one by one. This happened by iteratively investigating the identified questions using all datasets (i.e., following a thread) with an inductive approach. Through this process, we created a data repertoire to answer our research question. In the third phase, the analysis was deepened and refined as we synthesised all threads to answer the original research question by formulating new themes and writing up full descriptions of them.

### 2.5 Ethical Considerations

We conducted the study in alignment with the ethical guidelines provided by the Finnish National Board on Research Integrity (TENK) for research involving human participants (TENK 2019). Ethical clearance was secured from the University Ethics Committee (TY/40/12.12.2022, TY/131/06.01.01/2024, TY/699/06.01.01/2023). A research permit was obtained from the organisation (2023‐001224 T 13 02 01). We informed participants about the project in writing and orally about the aim, process, possible harms and benefits of the study, voluntary participation, participants' rights, restrictions to these. We also informed the participants about handling their personal data, which were processed in compliance with pseudonymisation or anonymisation protocols, as per Finnish (Data Protection Act 1050/2018) and European Union regulations (General Data Protection Regulation, Articles 13 and 14 2016). Informed consent from the participants was obtained in writing. The assessment of older adults' capacity to provide informed consent was conducted by staff members familiar with their conditions. In the case of not being capable, the older adults' family members were informed and gave informed consent on behalf of the older adult. We reported the study using mixed‐methods reporting guidelines (O'Cathain et al. 2008).

## 3 Results

Several plans were made regarding older adults' physical activity. However, older adults' activity levels remained low and organised physical activities were few. Older adults were mostly active in activities of daily living if supported by nurses, and independently if capable of independent ambulation. To answer the research question, we developed four themes: (1) lack of physical activity, (2) plans for physical activity promotion, (3) nurses' role in activity promotion and (4) accessibility and freedom of movement.

### 3.1 Lack of Physical Activity

Residents' physical activity levels were low and physical activity was of light intensity (Table 3). Only a small portion of physical activity was dedicated to organised activities. Measured with actigraphy (Fibion SENS Motion), older adults' physical activity was, on average, 1.27 h (76 min) per resident per day. This ranged from 0.00 h (0.16 min) to 4.37 h (262 min). Most of the average daily activity per resident consisted of standing (26 min), sporadic walking (29 min) and continuous walking (21 min). Moderate and vigorous intensity activity was observed only for one resident, being ≤ 6 min per day. The average physical activity of those who walked independently with or without an aid (*n* = 8) was 1.98 h (119 min) per day, including standing (40 min), sporadic walking (45 min) and continuous walking (34 min). The average activity of older adults who did not walk independently (*n* = 5) was 8 min per day. For one resident, the actigraphy did not record physical activity, as he/she did not use the lower body for movement.

**TABLE 3:** Older adults' (n=13) physical activity during 1 week measured with actigraphy.

Activity type minutes/day (*n* = 13)	Min	Max	Mean	SD
Standing	0.00	66.55	26.03	24.64
Sporadic walking	0.00	76.50	28.66	27.60
Continuous walking	0.00	129.62	20.89	34.48
**Light activity altogether, minutes/day**	**0.16**	**262.46**	**75.58**	**78.48**
Fast walking	0.00	4.41	0.34	1.22
Cycling	0.00	1.43	0.11	0.40
Vigorous activity	0.00	0.00	0.00	0.00
**Moderate and vigorous activity altogether, minutes/day**	**0.00**	**5.85**	**0.45**	**1.62**
**Activity altogether, minutes/day**	**0.16**	**262.46**	**76.03**	**78.93**
**Activity altogether, hours/day**	**0.00**	**4.37**	**1.27**	**1.32**

*Note:* Our analysis is descriptive in nature; therefore, inferential statistics (e.g., significance tests) were not applicable, as no comparisons or hypotheses were tested.

A total of 36 activities were organised for the older adults by the staff during the week. Family members visited the unit 20 times (Table 4). The staff organising the activities included nursing staff (including registered nurses, practical nurses and care assistants), a physiotherapist, an occupational therapist, a social worker and a cultural instructor.

**TABLE 4:** Older adults' organised activities for 1 week according to patient record transcripts.

Activity type	*N*	Min	Max	Mean	Median	Content of activity (when known)
Activities organised by nursing staff	13 (7*)	0	3	1.00	1	Physical activity (*n* = 7):* Walking exercise in hallway (*n* = 4) Playing ball games (*n* = 2) Visit to indoor garden (*n* = 1) Other activities (*n* = 6): Participation in the arts group (*n* = 2) Socialisation with a nurse (*n* = 2) Visit to indoor garden by wheelchair (*n* = 1) Musical activity (*n* = 1)
Physiotherapy	5 (5*)	0	2	0.38	0	Exercise (*n* = 5)* Stair exercise (*n* = 2) Riding a restorator ergometer (*n* = 2) Walking exercise (*n* = 1)
Occupational therapy	5 (1*)	0	1	0.4	0	Plant care in group (*n* = 4) Delivering goods in the facilities (*n* = 1)*
Activities organised by social worker	4	0	1	0.31	0	Musical group, singing
Activities organised by cultural instructor	2	0	1	0.15	0	Groups and events organised by the cultural instructor
Going outdoors	7 (5*)	0	2	0.54	0	Walking with a staff member (*n* = 5)* By wheelchair with a staff member (*n* = 2)
**Activities by staff, altogether**	**36 (** * **18**)	**0**	**8**	**2.77**	**3**	
Family visits	20 (4*)	0	5	1.54	1	Walking with a family member (*n* = 4)*
**Physical activities, altogether**	**22**	**0**	**5**	**1.69**	**2**	

*Note:* Our analysis is descriptive in nature; therefore, inferential statistics (e.g., significance tests) were not applicable, as no comparisons or hypotheses were tested.

* Physical activity.

Of the weekly activities organised for the residents by the staff (*n* = 36), half were physical activities (*n* = 18). The physical activities were primarily organised by the nursing staff and the physiotherapist, who organised 94% of the physical activities, including going outdoors. The physiotherapist's role was directly related to promoting physical activity.

…the physiotherapist, its more direct, like in their plan the goals directly relate to physical activity and promoting activity, so maybe, maybe like it's a bigger role, like in promoting physical activity. (staff member)

The occupational therapist's work focused more broadly on supporting all aspects of older adults' functioning through various activities. The social worker and cultural instructor organised activities and events that were passive, such as watching different performances.

Nine out of 13 residents (69%) participated in the physical activities organised by the staff. Of the older adults walking independently with or without an aid, seven out of eight (87.5%) participated in physical activities. Of those who ambulated independently with a wheelchair or required assistance from a staff member to move, two out of five participated in physical activities (40%). For those who participated in the physical activities, the organised physical activities accounted for, on average, 7 min per resident per day (Table 5), which was about 7% of their average total activity.

**TABLE 5:** Older adults' physical activity produced by organised physical activities for 1 week.

Physical activities for older adults	f	Duration	Altogether
Physiotherapy	5	25 min*	125 min
Activities by nurses	7	10 min**	70 min
Going outdoors and physically active occupational therapy	10	25 min***	250 min
**Altogether**			**445 min/week**
Physical activity produced by organised physical activities/older adult taking part (*n* = 9)			49 min/week 7 min/day
Actigraphy measured weekly physical activity/older adult taking part in organised activities (*n* = 9)			666 min/week 95 min/day
**Proportion of physical activity produced by physical activities**			**7%**

*Note:* Our analysis is descriptive in nature; therefore, inferential statistics (e.g., significance tests) were not applicable, as no comparisons or hypotheses were tested.

Abbreviation: f = frequency.

* Duration based on transcripts.

** Duration estimated based on content description of transcripts.

*** Duration estimated by staff working in the unit.

Family members visited the unit 20 times during the week, visiting eight different residents. They took two residents outdoors a total of four times during their visits. Based on the interview and focus group data, most family visits involved passive activities.

### 3.2 Plans for Physical Activity Promotion

The promotion of older adults' physical activity was guided by several plans and agreements made with older adults. These included the care plan, physical activity agreement, physiotherapy plan, occupational therapy plan and weekly physical activity plan. Providing needed support and guidance with a rehabilitative approach for older adults to perform activities of daily living was defined in the care plans. To inform them, the functional ability of each older adult was assessed in the activities of daily living by the nursing staff and the physiotherapist. Additionally, the physiotherapist conducted various measurements to evaluate functional ability. As a part of the care plan, a separate physical activity agreement was made for the residents, aiming to establish an agreement between the older adult and the care unit regarding physical activity, integrating it into daily activities and exercise and setting goals tailored to the individual's daily life.

Physiotherapy and occupational therapy plans were made for some residents assessed to have rehabilitation potential, ensuring the allocation of limited resources to the right individuals. Additionally, most older adults had a weekly physical activity plan consisting of short physical activities (2–5 times per week) and going outdoors once a week. The weekly physical activity plans were composed by the physiotherapist with the aim of increasing the physical activity of older adults. The delivery of activities according to the weekly plans was primarily the responsibility of the nursing staff; however, they did not have any specific time allocated for their delivery in the daily schedule. Some activities of the weekly physical activity plans were the shared responsibility of the nursing staff and the physiotherapist, such as going outdoors. Activities in accordance with the physiotherapy plan were also presented in the weekly physical activity plans for those receiving physiotherapy. The weekly physical activity plans were not intended to be rigid but to provide tools for the nursing staff to easily promote physical activity.

…they are like options out of what, what to choose from. So that you don't have to invent, what it would be like, that fifteen‐minute form of activity. (staff member)

The activities in the weekly physical activity plans included walking, stair walking, riding a restorator ergometer and exercising on a chair and were the same whether conducted by the nursing staff or as physiotherapy. However, the physiotherapy activities were longer in duration.

### 3.3 Nurses' Role in Activity Promotion

Based on the data, the promotion of activity by the nursing staff primarily occurred during activities of daily living. The residents participated in instrumental activities of daily living only occasionally and mostly with the occupational therapist. Nursing staff promoted the residents' physical activity with a function‐focused care approach in basic care, supporting the residents' independence in activities of daily living, thus promoting their physical activity. This required considering the residents' abilities, encouraging, guiding and being present.

you have to give time to them … then they do independently. (nurse)

As the nursing staff spent most of their time with the older adults and constituted the majority of the staff, their contribution to promoting the residents' physical activity was considered the most significant.

…is it doing for, or encouraging, or guiding, how care is provided. So that we get the residents like do things themselves, and we don't do for, it's like, it's like the key thing. That nurses adopt this kind of function focused care approach… (staff member)

Lack of time was reported as a barrier to implementing a function‐focused approach to care. In a rush, the residents' independence was not promoted as much as it could have been.

Of course it takes more time, if, well, you let the resident do him/herself. (nurse)

Lack of time was also considered to impede organising activities for the residents. According to the nursing staff themselves, their time was mostly spent on basic care tasks and other resident‐related duties, such as documenting, ordering supplies and managing medications. The nursing staff members organised 13 activities during the week, of which 7 were physical activities (54%). These included walking in the hallway (*n* = 4), playing ball games (*n* = 2) and visiting an indoor garden in the facilities (*n* = 1) (Table 3). Based on the descriptions, the activities were brief, such as walking from a resident's room to the dining room. The physiotherapist conducted almost as many physical activities (*n* = 5) as the nursing staff members during the week. In terms of time, the activities carried out by the physiotherapist (125 min) exceeded those conducted by the nursing staff members (70 min) (Table 5). Additionally, seven residents went outdoors with the staff during the week, two of whom did so passively in wheelchairs.

### 3.4 Accessibility and Freedom of Movement

Accessibility and freedom of movement inside the unit promoted independent activity of older adults. Based on the data, for residents capable of independent ambulation, a significant portion of physical activity consisted of independent activity. Comparing the duration of organised physical activities (approximately 7 min per day) to the average overall daily activity of those who walked independently (1.98 h per day), the amount of independent activity appeared relatively substantial, despite some of the activity occurring during activities of daily living. Based on the data, independent physical activity consisted of walking or ambulating with a wheelchair, as the data did not indicate any other physical activities that the residents engaged in independently. Aside from organised activities, leisure activities recorded for the residents included only passive activities, such as watching television and listening to the radio.

According to the participants, independent ambulation of the residents was facilitated by an accessible physical environment and freedom of movement in the unit. In the unit, the spacious, bright, recently renovated and accessible facilities enabled the residents to be physically active. The layout allowed the residents to go around the unit, aided by the fact that the in‐unit hallway doors were kept open during the daytime. However, the corridors were long, lacked places to sit and rest and did not have anything in them that would specifically stimulate moving around. Freedom of movement was rarely restricted by direct means, such as bed rails or wheelchair restraint belts. Instead, free movement was promoted through various practices.

We have a lot of residents that sleep on the floor on the mattress. They sleep being content on the mattress. Somehow they just pull themselves out of the bed there, they have this mattress next to them … it kind of makes movement possible during the night. (staff member)

Freedom of movement was limited to the unit because older adults were not allowed to exit it independently.

Well, you can't go outdoors, then there's the main door which is locked of course, so you have to go outdoors with a nurse or somebody else. (nurse)

Despite this, the unit's location within a facility with multiple services (e.g., restaurant, podiatrist, hair salon, library, gym, indoor garden, outdoor courtyard and easily accessible nature close by) was seen to promote physical activity. During the week, three older adults had visited the indoor facilities beyond their own unit with a staff member, and 11 visits had been made outdoors, of which four were with a family member.

## 4 Discussion

We formulated four themes about how, how much and by whom older adults' physical activity is promoted in institutional long‐term care. Based on our findings, older adults' activity levels were low and organised physical activities were few. Instead, older adults were mostly active in activities of daily living if supported by nurses and independently if capable of independent ambulation.

Older adults' low physical activity levels and activity conducted at a light intensity are consistent with previous studies reporting the physical activity levels of older adults (den Ouden et al. 2015; Hahn et al. 2023; Liu et al. 2020; Parry et al. 2019; Shi et al. 2024). This level is far behind the recommended levels for achieving the therapeutic effects of physical activity (de Souto Barreto et al. 2016; McArthur et al. 2024; Peyrusqué et al. 2023). Older adults were engaged in activities of daily living and mobility and in passive and organised activities. Physical activity was mostly produced in activities of daily living and mobility within the facilities, consistent with previous evidence (den Ouden et al. 2015; Parry et al. 2019). Passive activities included watching TV and listening to the radio, which have been reported in other studies (den Ouden et al. 2015), as older adults have nothing else to do (Narsakka et al. 2023). In our study, instrumental activities of daily living were not used to activate residents, which has also been reported in other studies (Parry et al. 2019). These findings point to the missed opportunities to meet care needs for older adults who require support to move and engage in activity.

Organised activities produced only 7% of the physical activity of the residents, despite the fact that compared to previous findings in Finland, activities were organised more for residents (Narsakka et al. 2023) and more residents participated in activities than in Finnish institutional long‐term care units (Edgren et al. 2021). This could relate to the fact that the unit's staff profile was more heterogeneous compared to the average (Kehusmaa and Alastalo 2021), which can be considered positive as interprofessional work in the institutional setting relates to high‐quality care (Doornebosch et al. 2022). However, only half of the provided activities were physical—mostly organised by the physiotherapist and the nurses. One opportunity to increase older adults' physical activity levels could be to include physical activity to some extent in all organised activities. Furthermore, combining physical and cognitive activities has benefits, such as improving cognitive functioning, performance in activities of daily living, and the mood of persons with dementia (Karssemeijer et al. 2017).

In our study, the participants identified nurses as the most important professionals for older adults' activity promotion. This has also been argued for in previous studies (Narsakka et al. 2023; Wylie et al. 2023). Furthermore, it is supported by the fact that activity should be conducted throughout the day (de Souto Barreto et al. 2016; Peyrusqué et al. 2023) and that nurses spend most of their time with residents. Also, as on average 2% of staff in Finland are rehabilitation professionals, their time alone is not sufficient to promote older adults' activity on a level that promotes their health and functioning. In the present study, the physiotherapist organised most of the physical activity. Nurses' activity promotion took place mostly during activities of daily living. Approaches integrating physical activity into care situations have been observed to lead to increased activity in older adults and could also reduce behavioural symptoms (Galik et al. 2021); however, evidence of their effectiveness is unclear (Hirt et al. 2024). Therefore, we argue that nurses should promote physical activity using multiple strategies.

To promote activity, nurses can use both spontaneous and planned activities. Lack of time is a commonly reported impediment to activity promotion by nurses (Hirt et al. 2024; Narsakka et al. 2022), as was the case in our study. Spontaneous activities by nurses can be used without a high demand for resources (Hahn et al. 2023). This could mean, for example, activating the residents during meal times, conducting small exercises after transfers, or dancing in between watching TV. Organised activities, on the other hand, are important for residents' well‐being (Peyrusqué et al. 2023). To incorporate these into daily practice, daily routines should include allocated time for activity promotion. This requires support from organisational and managerial levels.

In this study, there were many plans in use to guide physical activity promotion. However, the nurses did not have time specifically allocated for physical activities, and the extent to which older adults' weekly physical activity plans were executed remained low. A care culture promoting passivity can be deeply rooted in institutional care settings (Bowes et al. 2022), and nurses have been reported not to perceive the activity of older adults or its promotion as necessary or a part of their role (Narsakka et al. 2022). In this study, the physiotherapist composed weekly physical activity plans. A strategy to improve the implementation of new activities could include the nursing staff members themselves in developing their own practices (Bowes et al. 2022). This kind of approach could also affect care culture by improving staff engagement, teamwork and communication (Etherton‐Beer et al. 2013). Moreover, a lack of knowledge about the positive effects of physical activity for older adults has been identified as impeding activity promotion (Hahn et al. 2023). The education of nurses should include more knowledge and training about different strategies for physical activity promotion. Also, continuous education and educational interventions could be developed to improve activity promotion.

The physical environment promoted older adults' physical activity by being accessible and providing opportunities for independent walking, which have been identified as important in earlier research (Narsakka et al. 2022; Nordin et al. 2017) and seem to be considered in an increasing manner in long‐term care units (Narsakka et al. 2023). However, the positive benefits of having stimulating features in the physical environment (Narsakka et al. 2022) and access to outdoor spaces and indoor services beyond ones' living unit do not seem to be considered in our study or previous research (Narsakka et al. 2023). In the future, solutions to provide care in units where residents can have independent access beyond their living units should be studied and implemented in practice. Safety for this can be improved by technological solutions (Daly Lynn et al. 2019) and design features (Nordin et al. 2017). A very positive result in our study was that in addition to restricting the residents' freedom of movement by locked units, other restrictions on freedom of movement were used in a limited manner.

It could be stated that despite efforts to activate the residents, physical activity promotion remained limited in the present study. We recommend that in future practice, organisations should engage in systematic evaluations of their practices and the environment to meet a sufficient level in promoting older adults' activity. Evaluative practice could be improved by tools developed for this purpose. For example, a tool exists for the assessment of the physical environment in support of the independent functioning of residents (Wahlroos et al. 2021). New tools should be developed specifically to assess activity promotion in the institutional long‐term care setting. For organisations and professionals to incorporate physical activity as an integral part of daily life, various strategies could be used to physically activate older adults. Clinical nurse specialists could be employed to improve the implementation of existing evidence. Furthermore, actions should be taken to involve family members and volunteers in everyday activity promotion. This could possibly produce low‐cost solutions and have other benefits, such as improved quality of life and quality of care for older adults (Gaugler and Mitchell 2022).

Stimulating elements in the physical environment and increased freedom of movement could produce improvements in physical activity, and require both practice and policy changes to be used in delivering long‐term care. In future research, multicomponent physical activity interventions that can be modified to heterogeneous populations and contexts should be developed and tested to improve activity promotion. These interventions should be tested with older adults with varying functional impairments. Considering that culture is foundational to all social life, the actions of individuals and organisations (Geertz 1973) and the manifestations of the physical environment (Zimmerman et al. 2014), more focus should be given in future practice and research to changing the care culture for improved activity promotion in long‐term care settings. This requires the involvement of older adults and their carers.

### 4.1 Strengths and Limitations

Our study has many strengths. We involved older adults despite their physical and cognitive functioning, thus representing the population living in these settings. Patient record transcripts provided a less used means of evaluating physical activity and its promotion. As older adults' activity promotion largely depends on care professionals, investigating their perspectives, together with objectively measured physical activity and patient record transcripts of older adults, provides a means to produce an in‐depth understanding of the current situation of activity promotion.

The study also has limitations. The research was conducted as a single case study with a small sample of older adults, possibly limiting the generalisation of the results. In addition, the actigraphy used was suited only to measuring the activity produced with the lower body, limiting the measurement of the activity of older adults being physically active only or mostly with their upper bodies. However, these individuals represented a minority of our participants. Although patient record transcripts should include all information on the conducted activities, it could be possible that some information during the week was not recorded by the staff and, thus, was not available to us. Another limitation of our study was that older adults' own experiences and perspectives were not explored to answer the research question.

## 5 Conclusion

Our study produced new knowledge about older adults' physical activity promotion in an institutional long‐term care setting. Consistent with earlier evidence, older adults' activity levels are low and activity is conducted at a light intensity. For older adults to achieve benefits for their health and functioning, physical activity promotion should be improved. Improvements are needed in meeting plans to promote older adults' activity and to deliver sufficient physical activity. This can potentially be done with simple strategies and low additional costs. In addition to delivering recommended levels of exercise, activity promotion could be improved by various strategies that incorporate different professionals. To promote activity, nurses could use care‐integrated activities and spontaneous and organised activities to engage older adults in the instrumental activities of daily living. Interprofessional work to promote activity could be used more. Stimulating elements in the physical environment and increasing freedom of movement could result in improvements in physical activity. Considering the advanced decline in the cognitive and physical functioning of older adults living in the institutional long‐term care setting, physical activity promotion by care and the care environment is vital.

## Author Contributions

**Noora Narsakka:** conceptualisation, methodology, investigation, formal analysis, writing – original draft, writing – review and editing, visualisation, funding acquisition; **Riitta Suhonen:** conceptualisation, methodology, writing – review and editing, supervision, funding acquisition; **Johanna Finskas:** investigation, writing – review and editing; **Minna Stolt:** conceptualisation, methodology, writing – review and editing, supervision, funding acquisition.

## Ethics Statement

Ethical clearance was secured from the Ethics Committee for Human Sciences at the University of Turku, Health Care Division prior to initiating the study (40/2022/12.12.2022) and two subsequent amendments were approved during the project (TY/699/06.01.01/2023; TY/131/06.01.01/2024).

## Consent

Participants were informed in writing and orally about the aim, process and possible harms and benefits of the study, voluntary participation, participants' rights and restrictions to these, as well as handling their personal data. Informed consent was obtained in writing. In the case of not being capable of providing informed consent, older adults' family members were informed and gave informed consent on behalf of the older adult.

## Conflicts of Interest

Noora Narsakka, Riitta Suhonen and Minna Stolt do not have any conflicts of interest to declare. Johanna Finskas worked as a research assistant for the project and was also employed by the organisation where the study was conducted.

## Data Availability

Research data are not shared.

## References

[opn70053-bib-0001] Baldelli, G. , M. De Santi , F. De Felice , and G. Brandi . 2021. “Physical Activity Interventions to Improve the Quality of Life of Older Adults Living in Residential Care Facilities: A Systematic Review.” Geriatric Nursing 42, no. 4: 806–815. 10.1016/j.gerinurse.2021.04.011. 34090224

[opn70053-bib-0002] Barker, R. O. , B. Hanratty , A. Kingston , S. Ramsay , and F. E. Matthews . 2020. “Changes in Health and Functioning of Care Home Residents Over Two Decades: What Can We Learn From Population‐Based Studies?” Age and Ageing 50, no. 3: 921–927. 10.1093/ageing/afaa227. PMC8099147 33951152

[opn70053-bib-0003] Benjamin, K. , N. Edwards , J. Ploeg , and F. Legault . 2014. “Barriers to Physical Activity and Restorative Care for Residents in Long‐Term Care: A Review of the Literature.” Journal of Aging and Physical Activity 22, no. 1: 154–165. 10.1123/japa.2012-0139. 23434919

[opn70053-bib-0004] Bowes, A. , A. Dawson , C. Greasley‐Adams , R. Jepson , and L. McCabe . 2022. “Care Home Residents on the Move: The Significance of Cultural Context for Physical Activity.” Ageing and Society 42, no. 8: 1899–1920. 10.1017/S0144686X20001920.

[opn70053-bib-0005] Braun, V. , and V. Clarke . 2022. “Thematic Analysis: A Practical Guide.” QMiP Bulletin 1: 46–50.

[opn70053-bib-0006] Caspersen, C. J. , K. E. Powell , and G. Christenson . 1985. “Physical Activity, Exercise, and Physical Fitness: Definitions and Distinctions for Health‐Related Research.” Public Health Reports 100, no. 2: 126–131. 3920711 PMC1424733

[opn70053-bib-0007] Creswell, J. W. , and V. L. Plano Clark . 2018. Designing and Conducting Mixed Methods Research. Sage.

[opn70053-bib-0008] Cunningham, C. , R. O'Sullivan , P. Caserotti , and M. A. Tully . 2020. “Consequences of Physical Inactivity in Older Adults: A Systematic Review of Reviews and Meta‐Analyses.” Scandinavian Journal of Medicine & Science in Sports 30, no. 5: 816–827. 10.1111/sms.13616. 32020713

[opn70053-bib-0009] Daly Lynn, J. , J. Rondón‐Sulbarán , E. Quinn , A. Ryan , B. McCormack , and S. Martin . 2019. “A Systematic Review of Electronic Assistive Technology Within Supporting Living Environments for People With Dementia.” Dementia 18, no. 7: 2371–2435. 10.1177/1471301217733649. 28990408

[opn70053-bib-0010] Data Protection Act 1050/2018 . https://www.finlex.fi/fi/laki/alkup/2018/20181050.

[opn70053-bib-0011] de Souto Barreto, P. , J. E. Morley , W. Chodzko‐Zajko , et al. 2016. “Recommendations on Physical Activity and Exercise for Older Adults Living in Long‐Term Care Facilities: A Taskforce Report.” Journal of the American Medical Directors Association 17, no. 5: 381–392. 10.1016/j.jamda.2016.01.021. 27012368

[opn70053-bib-0012] den Ouden, M. , M. H. C. Bleijlevens , J. M. M. Meijers , et al. 2015. “Daily (In)activities of Nursing Home Residents in Their Wards: An Observation Study.” Journal of the American Medical Directors Association 16, no. 11: 963–968. 10.1016/j.jamda.2015.05.016. 26155723

[opn70053-bib-0013] Doornebosch, A. J. , H. J. A. Smaling , and W. P. Achterberg . 2022. “Interprofessional Collaboration in Long‐Term Care and Rehabilitation: A Systematic Review.” Journal of the American Medical Directors Association 23, no. 5: 764–777. 10.1016/j.jamda.2021.12.028. 35065048

[opn70053-bib-0014] Edgren, J. , J. Asikainen , J. Häsä , and M. Aaltonen . 2024. “Iäkkäiden Toimintakyky ja Palvelutarpeet—RAI‐Vertailutiedot 2022 [Functioning and Service Needs of Older People—RAI Benchmarking Data 2022].” TILASTORAPORTTI 3/2024.

[opn70053-bib-0015] Edgren, J. , L. Penttinen , M. Mäkelä , J. Asikainen , A. Gerasin , and S. Havulinna . 2021. “Ikääntyneen Asiakkaan Kuntoutumisen Voimavarat Jäävät Usein Hyödyntämättä [Older Customer's Rehabilitation Resources Are Often Left Unexploited].” Tutkimuksesta Tiiviisti [Research Results in Short] 46/2021. Finnish National Institute for Health and Welfare. https://urn.fi/URN:ISBN:978‐952‐343‐703‐6.

[opn70053-bib-0016] Etherton‐Beer, C. , L. Venturato , and B. Horner . 2013. “Organisational Culture in Residential Aged Care Facilities: A Cross‐Sectional Observational Study.” PLoS One 8, no. 3: e58002. 10.1371/journal.pone.0058002. 23505450 PMC3591446

[opn70053-bib-0017] Fibion Inc . 2020. “Fibion SENS.” https://sens.fibion.com/.

[opn70053-bib-0018] Finnish National Board on Research Integrity TENK . 2019. “The Ethical Principles of Research With Human Participants and Ethical Review in the Human Sciences in Finland: Finnish National Board on Research Integrity TENK Guidelines 2019.” Publications of the Finnish National Board on Research Integrity TENK 3/2019. Finnish National Board on Research Integrity TENK, Helsinki.

[opn70053-bib-0019] Galik, E. M. , B. Resnick , S. D. Holmes , et al. 2021. “A Cluster Randomized Controlled Trial Testing the Impact of Function and Behavior Focused Care for Nursing Home Residents With Dementia.” Journal of the American Medical Directors Association 22, no. 7: 1421–1428. 10.1016/j.jamda.2020.12.020. 33454311 PMC8280250

[opn70053-bib-0020] Gaugler, J. E. , and L. L. Mitchell . 2022. “Reimagining Family Involvement in Residential Long‐Term Care.” Journal of the American Medical Directors Association 23, no. 2: 235–240. 10.1016/j.jamda.2021.12.022. 34973167 PMC8821144

[opn70053-bib-0021] Geertz, C. 1973. The Interpretation of Cultures: Selected Essays. Basic Books.

[opn70053-bib-0022] General Data Protection Regulation 2016/679, Articles 13 and 14 .

[opn70053-bib-0023] Hahn, L. S. , A. Thiel , D. Trüb , et al. 2023. “Patterns of Physical Activity Among Nursing Home Residents Before and During the Covid 19 Pandemic—A Systematic Observation.” European Review of Aging and Physical Activity 20, no. 1: 23–28. 10.1186/s11556-023-00332-5. 38057739 PMC10702021

[opn70053-bib-0024] Hirt, J. , J. Vetsch , I. Weissenfels , and S. Heinrich . 2024. “Nurse‐Led Physical Activity Interventions for People With Dementia in Nursing Homes: A Systematic Review on Intervention Characteristics and Implementation Facilitators/Barriers.” International Journal of Nursing Studies 154: 104756. 10.1016/j.ijnurstu.2024.104756. 38552471

[opn70053-bib-0025] Jepson, R. G. , A. Dawson , L. McCabe , C. Greasley‐Adams , H. Biggs , and A. Bowes . 2023. “Development of an Intervention Programme Theory to Increase Movement in Care Homes for People With Cognitive Impairment: Care Homes Achieving Realistic Movement Strategies (CHARMS).” Evaluation and Program Planning 100: 102348. 10.1016/j.evalprogplan.2023.102348. 37506615

[opn70053-bib-0026] Johnson, R. B. , A. J. Onwuegbuzie , and L. A. Turner . 2007. “Toward a Definition of Mixed Methods Research.” Journal of Mixed Methods Research 1, no. 2: 112–133. 10.1177/1558689806298224.

[opn70053-bib-0027] Karssemeijer, E. G. A. , J. A. Aaronson , W. J. Bossers , T. Smits , M. G. M. Olde Rikkert , and R. P. C. Kessels . 2017. “Positive Effects of Combined Cognitive and Physical Exercise Training on Cognitive Function in Older Adults With Mild Cognitive Impairment or Dementia: A Meta‐Analysis.” Ageing Research Reviews 40: 75–83. 10.1016/j.arr.2017.09.003. 28912076

[opn70053-bib-0028] Kehusmaa, S. , and H. Alastalo . 2021. “Lähi‐ ja Sairaanhoitajien Määrä ei Vielä Ole Noussut Vanhuspalveluissa [Number of Practical and Registered Nurses Has Not Risen yet in Elderly Care Services].” THL – Tutkimuksesta Tiiviisti [Research Results in Short] 47/2021 Finnish Institute for Health and Welfare. https://urn.fi/URN:ISBN:978‐952‐343‐704‐3.

[opn70053-bib-0029] Kim, H. S. 2010. The Nature of Theoretical Thinking in Nursing. Springer Pub. Co.

[opn70053-bib-0030] Lane, N. E. , W. P. Wodchis , C. M. Boyd , and T. A. Stukel . 2017. “Disability in Long‐Term Care Residents Explained by Prevalent Geriatric Syndromes, Not Long‐Term Care Home Characteristics: A Cross‐Sectional Study.” BMC Geriatrics 17, no. 1: 49–79. 10.1186/s12877-017-0444-1. 28183274 PMC5301427

[opn70053-bib-0031] Lawton, M. P. 1989. “Three Functions of the Residential Environment.” Journal of Housing for the Elderly 5, no. 1: 35–50.

[opn70053-bib-0032] Liu, L. , H. Liu , M. Xiang , H. Guo , Z. Sun , and T. Wu . 2020. “Prevalence and Associated Factors of Physical Activity Among Older Adults Living in Nursing Homes: A Cross‐Sectional Study.” Journal of Gerontological Nursing 46, no. 10: 19–26. 10.3928/00989134-20200706-01. 32640032

[opn70053-bib-0033] Maurer, C. , S. Draganescu , H. Mayer , and H. Gattinger . 2019. “Attitudes and Needs of Residents in Long‐Term Care Facilities Regarding Physical Activity—A Systematic Review and Synthesis of Qualitative Studies.” Journal of Clinical Nursing 28, no. 13: 2386–2400. 10.1111/jocn.14761. 30589972

[opn70053-bib-0034] Mc Ardle, R. , K. Sverdrup , S. Del Din , et al. 2021. “Quantifying Physical Activity in Aged Residential Care Facilities: A Structured Review.” Ageing Research Reviews 67: 101298. 10.1016/j.arr.2021.101298. 33592308

[opn70053-bib-0035] McArthur, C. , N. Alizadehsaravi , R. Affoo , et al. 2024. “Effectiveness of Physical Rehabilitation on Physical Functioning and Quality of Life for Long‐Term Care Residents With Dementia: A Systematic Review and Meta‐Analysis.” JBI Evidence Synthesis 22: 1460–1535. 10.11124/JBIES-23-00431. 38915237 PMC11321609

[opn70053-bib-0036] McArthur, C. , J. C. Gibbs , R. Patel , et al. 2017. “A Scoping Review of Physical Rehabilitation in Long‐Term Care: Interventions, Outcomes, Tools.” Canadian Journal on Aging 36, no. 4: 435–452. 10.1017/S071498081700040X. 29130428

[opn70053-bib-0037] Mielikäinen, L. , and M. Kuronen . 2022. “Sosiaalihuollon Laitos‐ ja Asumispalvelut 2021: Sosiaalihuollon Ympärivuorokautisissa Laitos‐ ja Asumispalveluissa Vuoden Aikana yli 100 000 Asiakasta [Social Care Institutional and Housing Services in 2021: More Than 100 000 Customers Lived in Social Care Institutional and Housing Services].” THL – Tilastoraportti [Statistical Report] 26/2022. Finnish Institute for Health and Welfare. https://www.julkari.fi/bitstream/handle/10024/144576/Sosiaalihuollon%20laitos‐%20ja%20asumispalvelut_2021.pdf?sequence=1&isAllowed=y.

[opn70053-bib-0052] Moran‐Ellis, J. , V. D. Alexander , A. Cronin , et al. 2006. “Triangulation and Integration: Processes, Claims and Implications.” Qualitative Research: QR 6, no. 1: 45–59. 10.1177/1468794106058870.

[opn70053-bib-0038] Narsakka, N. , R. Suhonen , B. Groot , and M. Stolt . 2023. “Promoting Activity and Mobility in Long‐Term Care Environments: A Photo‐Elicitation Study With Older Adults and Nurses.” Journal of Clinical Nursing 32: 23–24. 10.1111/jocn.16866. 37698144

[opn70053-bib-0039] Narsakka, N. , R. Suhonen , E. Kielo‐Viljamaa , and M. Stolt . 2022. “Physical, Social, and Symbolic Environment Related to Physical Activity of Older Individuals in Long‐Term Care: A Mixed‐Method Systematic Review.” International Journal of Nursing Studies 135: 104350. 10.1016/j.ijnurstu.2022.104350. 36116327

[opn70053-bib-0040] Nordin, S. , K. McKee , M. Wallinder , L. von Koch , H. Wijk , and M. Elf . 2017. “The Physical Environment, Activity and Interaction in Residential Care Facilities for Older People: A Comparative Case Study.” Scandinavian Journal of Caring Sciences 31, no. 4: 727–738. 10.1111/scs.12391. 27862156

[opn70053-bib-0041] O'Cathain, A. , E. Murphy , and J. Nicholl . 2008. “The Quality of Mixed Methods Studies in Health Services Research.” Journal of Health Services Research & Policy 13, no. 2: 92–98. 10.1258/jhsrp.2007.007074. 18416914

[opn70053-bib-0042] OECD . 2021. Health at a Glance 2021: OECD Indicators. OECD Publishing. 10.1787/ae3016b9-en.

[opn70053-bib-0043] Palese, A. , G. Menegazzi , A. Tullio , M. Zigotti Fuso , M. Hayter , and R. Watson . 2016. “Functional Decline in Residents Living in Nursing Homes: A Systematic Review of the Literature.” Journal of the American Medical Directors Association 17, no. 8: 694–705. 10.1016/j.jamda.2016.04.002. 27233488

[opn70053-bib-0044] Parry, S. , M. Chow , F. Batchelor , and R. E. Fary . 2019. “Physical Activity and Sedentary Behaviour in a Residential Aged Care Facility.” Australasian Journal on Ageing 38, no. 1: E12–E18. 10.1111/ajag.12589. 30281184

[opn70053-bib-0045] Peyrusqué, E. , F. Buckinx , M.‐J. Kergoat , and M. Aubertin‐Leheudre . 2023. “Exercise Guidelines to Counteract Physical Deconditioning in Long‐Term Care Facilities: What to Do and How to Do It?” Journal of the American Medical Directors Association 24: 583–598. 10.1016/j.jamda.2023.01.015. 36822232

[opn70053-bib-0046] Shi, Y. , X. Xie , A. Lao , L. Shao , Z. Wang , and J. Zhang . 2024. “Prevalence of Physical Inactivity and Its Determinants Among Older Adults Living in Nursing Homes: A Cross‐Sectional Study Based on COM‐B Model.” Journal of Clinical Nursing 34: 204–217. 10.1111/jocn.17325. 38867609

[opn70053-bib-0047] Terveyden ja Hyvinvoinnin Laitos (THL) [National Institute for Health and Welfare] . 2024. “Sosiaalihuollon Laitos‐ ja Asumispalvelut [Social Care Institutional and Living Services 2023].” Tilastoraportti 22/2024 [Statistical Report 22/2024]. Suomen Virallinen Tilasto (SVT) [Finnish Official Statistics].

[opn70053-bib-0048] Wahlroos, N. , M. Stolt , S. Nordin , and R. Suhonen . 2021. “Evaluating Physical Environments for Older People—Validation of the Swedish Version of the Sheffield Care Environment Assessment Matrix for Use in Finnish Long‐Term Care.” International Journal of Older People Nursing 16: e12383. 10.1111/opn.12383. 34029003

[opn70053-bib-0049] World Health Organization and Alzheimer's Disease International . 2012. “Dementia: A Public Health Priority.” https://www.who.int/publications/i/item/dementia‐a‐public‐health‐priority.

[opn70053-bib-0050] Wylie, G. , T. Kroll , M. D. Witham , and J. Morris . 2023. “Increasing Physical Activity Levels in Care Homes for Older People: A Quantitative Scoping Review of Intervention Studies to Guide Future Research.” Disability and Rehabilitation 1: 1–17. 10.1080/09638288.2022.2118869. PMC10503503 36093619

[opn70053-bib-0051] Zimmerman, S. , V. Shier , and D. Saliba . 2014. “Transforming Nursing Home Culture: Evidence for Practice and Policy.” Gerontologist 54, no. 1: S1–S5. 10.1093/geront/gnt161. 24443601

